# Glioma and microenvironment dual targeted nanocarrier for improved antiglioblastoma efficacy

**DOI:** 10.1080/10717544.2017.1378940

**Published:** 2017-09-21

**Authors:** Xiuzhen Wang, Qing Zhang, Lingyan Lv, Junjie Fu, Yan Jiang, Hongliang Xin, Qizheng Yao

**Affiliations:** a Department of Medicinal Chemistry, School of Pharmacy, China Pharmaceutical University, Nanjing, China;; b School of Pharmacy, Nanjing Medical University, Nanjing, China

**Keywords:** Glioblastoma, blood–brain tumor barrier (BBTB), Pep-1, CREAK, targeting delivery system

## Abstract

Drug delivery systems based on nanoparticles (nano-DDS) have aroused attentions for the treatment of glioblastoma (GBM), the most malignant brain cancer with a dismal prognosis. However, there are still numerous unmet challenges for traditional nano-DDS, such as the poor nanoparticle penetration, short retention in the GBM parenchyma and low glioma targeting ability. Herein, we used Pep-1 and CREKA peptides to construct a novel multifunctional GBM targeting nano-DDS (PC-NP). Pep-1 was used to overcome the blood–brain tumor barrier (BBTB) and home to glioma cells via interleukin-13 receptor-α2-mediated endocytosis, and CREKA was used to bind to fibrin–fibronectin complexes abundantly expressed in tumor microenvironment for enhanced retention in the GBM. Biological studies showed that the cellular uptake of PC-NP by U87MG cells was significantly enhanced compared with the non-targeting NP. Furthermore, CREKA modification increased the binding capacity of PC-NP to fibrin–fibronectin complexes as confirmed by the competition experiment. In accordance with the increased cellular uptake, PC-NP remarkably increased the cytotoxicity of its payload paclitaxel (PTX) against U87MG cells with an IC_50_ of 0.176 μg/mL. *In vivo* fluorescence imaging and antiglioma efficacy evaluation further confirmed that PC-NP accumulated effectively and penetrated deeply into GBM tissue. PC-NP-PTX exhibited a median survival time as long as 61 days in intracranial GBM-bearing mice. In conclusion, our findings indicated PC-NP as a promising nano-DDS for GBM targeting delivery of anticancer drugs.

## Introduction

Glioblastoma (GBM), which is defined as grade IV by the World Health Organization (Fuller & Scheithauer, 2007), is the most common primary malignant brain tumor (Ostrom et al., 2014). It has a poor prognosis with a mean survival time of less than 18 months and a 5-year survival rate of less than 5% (Jo et al., 2012; Colombo et al., 2015). Unfortunately, complete surgical removal of glioma is limited due to the high invasiveness (Colombo et al., 2015). In most cases, GBM invariably recurs even when complete resections have been performed with existing pre-operative planning and post-operative imaging techniques. Moreover, the large majority of recurrences are at the resection cavity margin (Petrecca et al., 2013; Colombo et al., 2015). Therefore, chemotherapy is considered to be essential for GBM treatment (Chung et al., 2014). Currently, several chemotherapeutic drugs have been approved by the Food and Drug Administration (FDA) for the treatment of GBM (Bianco et al., 2017). However, there is still no significant improvement in median survival (Campos et al., 2016). The poor outcome is mainly ascribed to several physiologic barriers in glioma, such as blood–brain barrier (BBB) and the blood–brain tumor barrier (BBTB), which prevent the sufficient delivery of therapeutic agents into glioma tissue (Deeken & Loscher, 2007; Juillerat-Jeanneret, 2008; van Tellingen et al., 2015). In addition, poor glioma targeting and high-level of GMB chemoresistance also hinder the chemotherapeutic effects (Kim et al., 2015b).

In recent years, much nanodrug delivery systems (nano-DDS) modified with targeted ligands were developed for glioma treatment, such as liposomes (Chen et al., 2017; Zhang et al., 2017), micelles (Sonali et al., 2016), nanoparticles (Wang et al., 2014; Jiang et al., 2017), polymersomes (Lu et al., 2017) and dendrimers (Zhao et al., 2015). They are based on the ability of nano-DDS to increase the accumulation and maintain a high concentration of chemotherapeutic agents in tumors via the enhanced permeability and retention (EPR) effects (Perez-Herrero & Fernandez-Medarde, 2015) and then uptake into glioma cells through endocytosis. However, GBM is characterized by small microvascular pore size and sustained high interstitial fluid pressure (Boucher et al., 1990; Sarin et al., 2009), which compromise the tumor penetration and retention of nano-DDS, limiting the therapeutic effects (Kim et al., 2015a; Sehedic et al., 2015). To overcome the above problems, novel nano-DDS with improved capabilities to penetrate BBTB, retain at the GBM parenchyma and translocate drug into glioma cells is urgently required. Considering the complex glioma characterized by high heterogeneity, the use of a single targeted strategy might only be a suboptimal strategy. Thus, we developed glioma and microenvironment dual targeted nanocarrier through bi-ligand modification for improved anti-GBM efficacy.

In this work, Pep-1 and CREKA peptides were employed to modify the nano-DDS. Both of the two peptides were previously discovered by phage display screening (Simberg et al., 2007; Pandya et al., 2012). Pep-1 (Cys-Gly-Glu-Met-Gly-Trp-Val-Arg-Cys) functions as a BBTB-penetrating and glioma targeting peptide by specifically binding to interleukin-13 receptor-α2 (IL-13Ra2) with high affinity, followed by internalization into glioma cells (Pandya et al., 2012). IL-13Rα2, type I internalized plasma membrane receptor (Hershey, 2003), is abundantly expressed in established glioma cell lines and primary GBM cell cultures (Debinski et al., 1999; Brown et al., 2012; Pandya et al., 2012). These characteristics endow Pep-1 peptide with potential ‘dual targeting’ property of crossing the BBTB and homing to the glioma via IL-13Rα2-mediated endocytosis. Thus, Pep-1-modified nanoparticles are expected to achieve improved delivery of therapeutic agents to glioma, leading to enhanced antiglioma activity. The peptide CREKA (Cys-Arg-Glu-Lys-Ala) (Song et al., 2014), a targeting ligand for the fibrin–fibronectin complexes in the tumor extracellular matrix (ECM) (Simberg et al., 2007; Ye et al., 2008), works as an anchor to immobilize nanoparticles within the tumor interstitial to enhance retention in glioma tissue. The high expression of fibrin–fibronectin complexes, as the result of protein seepage of tumor vessels and the procoagulant effect in the tumor microenvironment (Abe et al., 1999; Wang et al., 2015), is related to many invasive and metastatic tumor phenotypes (Malik et al., 2010), including primary and metastatic brain tumors (Bardos et al., 1996). In fact, the fibrin–fibronectin complexes have been reported as biomarkers for the early detection and diagnosis of cancer and micrometastasis (Zhou et al., 2015).

**Figure 1.**
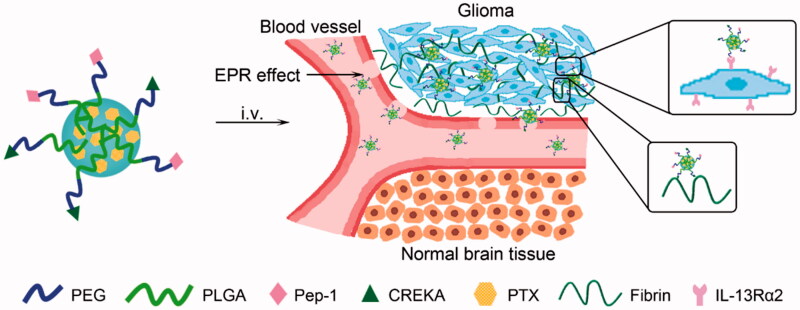
Design of Pep-1 and CREKA dual-conjugated PEG-PLGA nanocarrier (PC-NP) for glioma targeting delivery based on the strong binding capacity of CREKA to fibrin–fibronectin complexes and IL-13Rα2-mediated endocytosis.

Herein, we develop a peptide-mediated nano-DDS (PC-NP) by dual conjugations of Pep-1 and CREKA to the surface of PEG-PLGA nanoparticle. PEG-PLGA is used as a nanoscale carrier due to its good biocompatibility, biodegradability and stealth property in blood circulation (Yang et al., 2007). Pep-1 and CREKA dual modifications aim at crossing the BBTB, enhancing the retention at tumor site and improving the glioma targeting delivery of PEG-PLGA nanoparticle (Figure 1). In this nanoparticle system, PEG acts as the shell and PLGA acts as the core, into which hydrophobic model drugs [i.e. coumarin-6, DiR, paclitaxel (PTX)] can then be effectively encapsulated. In order to investigate the glioma targeting ability and antiglioma efficacy of PC-NP, coumarin-6 and DiR were used as probes, and PTX was used as the model hydrophobic drug.

## Materials and methods

### Materials

Pep-1 and CREKA peptides were synthesized by the GL Biochem Co., Ltd. (Shanghai, China). MePEG-PLGA and maleimide-PEG-PLGA were obtained from Daigang Biomaterial Co., Ltd. (Jinan, China). The synthesis and characterization of Pep-PEG-PLGA and CREKA-PEG-PLGA were described in the Supporting Information. PTX was purchased from Zelang Medical Technology Co., Ltd. (Nanjing, China). Coumarin-6 and DiR (1,1'-dioctadecyl-3,3,3',3'-tetramethyl indotricarbocyanine iodide) were provided by Sigma-Aldrich (St. Louis, MO). MTT (3-(4,5-dimethyl-2-thiazolyl)-2,5-diphenyl-2H-tetrazolium bromide), BCA kit and Triton X-100 were provided by Beyotime Biotechnology Co., Ltd. (Nantong, China). Penicillin–streptomycin, DMEM medium, fetal bovine serum (FBS) and trypsin solution were purchased from Gibco BRL (Gaithersburg, MD. All the other reagents were of analytical grade.

### Animals and cell line

BALB/c nude mice (male, 20 ± 2 g) were supplied by BK Lab Animal Ltd. (Shanghai, China) and housed at 25 ± 1 °C with free access to food and water. All animal experiments were performed in accordance with protocols evaluated and approved by the ethics committee of Nanjing Medical University.

U87MG cells (human malignant GBM cells) were obtained from Institute of Biochemistry and Cell Biology, Shanghai Institutes for Biological Sciences, Chinese Academy of Sciences (Shanghai, China). The cell line was cultured in DMEM medium and supplemented with 10% (v/v) FBS, 100 U/mL penicillin and 100 mg/mL streptomycin at 37 °C in a humidified atmosphere of 5% CO_2_. All experiments were performed in the logarithmic phase of cell growth.

### Preparation of Pep-NP, CREKA-NP and PC-NP

Pep-1- and CREKA-modified nanoparticle containing PTX (PC-NP-PTX) was prepared using emulsion/solvent evaporation technique. Briefly, the mixture of MePEG-PLGA copolymer (19 mg), Pep-PEG-PLGA (2 mg), CREKA-PEG-PLGA (2 mg) and PTX (1 mg) was dissolved in ethyl acetate (1 mL) and then added into 2 mL of poloxamer 188 aqueous solution (1% (w/v)). After pulse ultrasonication for 5 min using intermittent probe sonication (Xin Zhi Biotechnology Co., Ltd., JY92-IIDN, Ningbo, China) in an ice bath, the resultant O/W emulsion was immediately dispersed into 10 mL of poloxamer 188 aqueous solution (0.5% (w/v)) under stirring (650 rpm) for 5 min. The residual ethyl acetate was removed by a vacuum rotary evaporator at 40 °C. The obtained bluish solution was filtrated with 0.45- and 0.22-μm filters subsequently to separate the unentrapped drugs.

Pep-NP-PTX and CREKA-NP-PTX were prepared with the same procedure as above but in the absence of CREKA-PEG-PLGA or Pep-PEG-PLGA, respectively. In addition, for the preparation of fluorescently labeled nanoparticles, PTX was replaced with 0.2 mg of coumarin-6 or DiR.

### Characterization of nanoparticles

The morphology of PC-NP-PTX was characterized by transmission electronic microscopy (JEOL Ltd., JEM-1010, Tokyo, Japan). The diameter of PC-NP-PTX was measured by dynamic light scattering (Malvern Instruments Ltd., ZS90, Malvern, UK). The encapsulation efficiency (EE) and loading capacity (LC) of PTX in various nanoparticles were measured by HPLC (Agilent Technologies Inc., Agilent 1100, Palo Alto, CA) with UV-Vis detection. The EE% and LC% were calculated as indicated below (*n* = 3)

$$EE\left(\%\right)=\frac{Amount\;of\;PTX\;in\;nanoparticles}{Total\;amount\;of\;PTX\;added}\times 100\%$$

$$LC\left(\%\right)=\frac{Amount\;of\;PTX\;in\;nanoparticles}{Weight\;of\;nanoparticles}\times 100\%$$

### *In vitro* release of PTX from nanoparticles

*In vitro* release behaviors of PTX from nanoparticles were evaluated by dialysis method in PBS containing 0.5% (w/v) Tween 80 at different pH values (pH 7.4 and 5.0) at 37 °C. In brief, the PTX-loaded nanoparticles were diluted with release medium to a PTX concentration of 0.09 mg/mL. One milliliter of PTX-loaded NP, Pep-NP, CREKA-NP and PC-NP was tightly sealed in a dialysis bag (MWCO 7000 Da), respectively. Then, the bags were completely submerged in a centrifuge tube containing 40 mL of mediator solution and incubated in a shaking water bath (37 °C, 120 rpm). Aliquots (400 μL) were taken out and replenished immediately with equal amount of volume of the release medium at predetermined time. Finally, the amount of PTX released from the various nanoparticles was quantitatively measured by HPLC.

### Cellular uptake assay

For qualitative assay, U87MG cells were seeded in a 6-well plate at the density of 3 × 10^5^ cells per well for 24 h and then incubated with various coumarin-6-labeled nanoparticles at different concentrations (10, 20 and 40 ng/mL) for an additional 1 h. Subsequently, the cells were rinsed, fixed with 4% formaldehyde for 15 min and imaged by fluorescent microscopy.

To study the effect of nanoparticle concentration on cellular uptake, U87MG cells were incubated with the coumarin-6-labeled nanoparticles at different concentrations (200, 400, 600, 800 and 1000 ng/mL) for 1 h at 37 °C. To study the effect of incubation time on cellular uptake, U87MG cells were incubated with 500 ng/mL of coumarin-6-labeled nanoparticles at 37 °C for different incubation times (0.5, 1, 2, 3 and 4 h). After incubation, the cells were washed with pre-cooled PBS and lysed by 400 μL of 1% Triton X-100 per well for 10 min. The aliquot of the cell lysate was measured by using the BCA protein assay to determine the total cell protein content and the concentration of coumarin-6 was analyzed by HPLC to determine fluorescence intensity of each well.

### *In vitro* cytotoxicity assay

U87MG cells were seeded in a 96-well plate at the density of 5 × 10^3^ cells per well. After incubation for 24 h, the cells were treated with Taxol^®^, NP-PTX, Pep-NP-PTX, CREKA-NP-PTX and PC-NP-PTX for 48 h at different concentrations (from 0.001 to 10 μg/mL), respectively. Then, cells were exposed to 20 μL of MTT (5 mg/mL) for further 4 h. Afterwards, medium was gently replaced with 200 μL DMSO to dissolve the formazan crystals, and the cells were incubated for 15 min at room temperature in the absence of light. The cell viability was calculated from the absorbance measured with a microplate reader (Thermo Fisher Scientific, Multiskan MK3, Waltham, MA).

### *In vitro* fibrin clot binding assay

First, the *in vitro* fibrin clots were formed by the procedure as previously described (Zhao et al., 2015). Briefly, the fibrin clots were prepared by incubating 90 μL of fresh frozen plasma (FFP) with 5 μL of 0.4 M CaCl_2_ and 25 μL of thrombin (0.1 U/ml) in a 96-well plate at 37 °C for 90 min. Subsequently, 50 μL of DiR-labeled nanoparticles was added into each well. After incubation in dark at 37 °C for 30 min, the fibrin clots were washed twice using PBS and then centrifuged at 500 rpm for 10 min. Finally, after repeatedly washing with PBS, the samples were imaged and analyzed by the *in vitro* IVIS spectrum imaging system (PerkinElmer, Maestro, Waltham, MA).

For the competition inhibition assay, the free CREKA was added into the wells after clot formation with the concentration of CREKA adjusted to 500 μg/mL. After pre-incubation at 37 °C for 30 min, 50 μL of the DiR-labeled PC-NP (50 μL/mL) was added, followed by imaging analysis.

### *In vivo* imaging analysis

U87MG cells (2 × 10^5^ cells suspended in 5 μL of PBS) were slowly injected into the right corpus striatum (1.8 mm lateral, 0.6 mm anterior to the bregma and 3 mm of depth) of BALB/c nude mice using a stereotaxic apparatus. The intracranial glioma-bearing mice were randomly divided into four groups, which were injected intravenously via the tail vein with DiR-labeled NP, CREKA-NP, Pep-NP and PC-NP (0.1 mg/kg) 14 days post-inoculation, respectively. Then, the real-time imaging was performed at predetermined time points (4 and 24 h) using an *in vivo* imaging system (PerkinElmer, Maestro, Waltham, MA). Subsequently, the mice were sacrificed and the organs including brain, heart, liver, spleen, lung and kidney were harvested for *ex vivo* imaging.

For qualitative analysis of brain biodistribution of nanoparticles, 14 days after U87MG implantation, the glioma-bearing mice were tail intravenously injected with coumarin-6-labeled NP, Pep-NP, CRKEA-NP and PC-NP (0.2 mg/kg), respectively. Two hours after injection, the mice were anesthetized and subjected to perfusion with saline and 4% paraformaldehyde, respectively. After that, the brains were harvested, fixed in 4% paraformaldehyde, dehydrated with 15%, 30% sucrose solution and embedded in optimal cutting temperature (OCT) (Sakura, Torrance, CA) sequentially. Thereafter, each brain was cut into 5-μm slices by frozen section and examined with fluorescent microscopy after nuclei stained with DAPI.

### *In vivo anti-*GBM *efficacy*

U87MG GBM-bearing mice were divided into six groups. At the 2, 4, 6 and 8 days after U87MG cell inoculation, the mice were tail intravenously injected with saline, Taxol^®^, NP-PTX, Pep-NP-PTX, CREKA-NP-PTX and PC-NP-PTX at a PTX dose of 10 mg/kg, respectively. The survival of mice was estimated according to the Kaplan–Meier method and analyzed with a log-rank test.

### Statistical analysis

All the results were expressed as mean ± standard deviation (SD). One-way ANOVA was utilized for statistical evaluation. Statistical analysis was performed with SPSS 20.0 software (SPSS Inc., Chicago, IL).

## Results and discussion

### Characterization of nanoparticles

**Figure 2.**
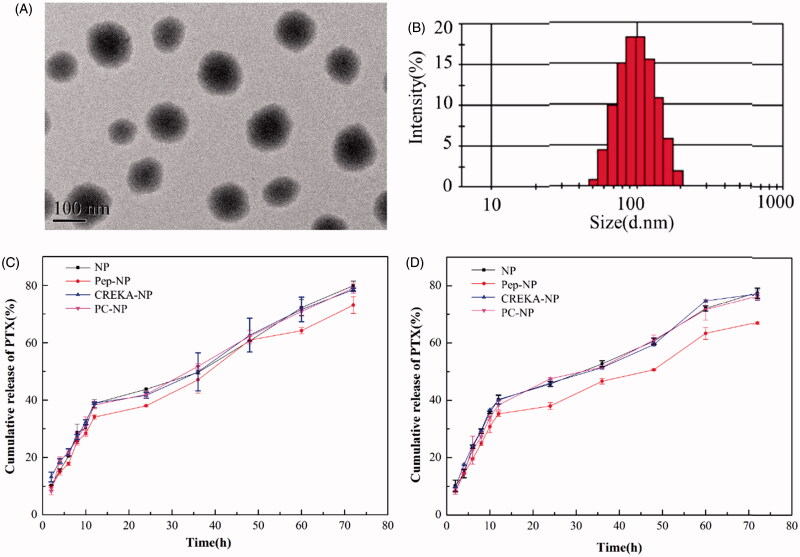
TEM image (A) and particle size distribution (B) of PC-NP-PTX. PTX release profiles of various nanoparticles in PBS (pH 5.0) (C) and PBS (pH 7.4) (D) with 0.5% (w/v) Tween 80.

The nanoparticles were prepared via an emulsion/solvent evaporation method and their physical characterizations including particle size, zeta potential, EE% and LC% were shown in Tables S1 (Supporting Information). The mean particle size of NP-PTX was 89.3 ± 1.2 nm. After modified with Pep-1 and/or CREKA peptide, the average diameter of Pep-NP-PTX, CREKA-NP-PTX and PC-NP-PTX slightly increased to 93.2 ± 2.4 nm, 95.8 ± 4.2 nm and 101.1 ± 2.8 nm, respectively. All the obtained nanoparticles exhibited narrow size distributions, with a zeta potential of −32.5 ± 1.2 mV, −33.6 ± 1.3 mV, −20.3 ± 3.1 mV and −25.6 ± 2.5 mV for NP-PTX, Pep-NP-PTX, CREKA-NP-PTX and PC-NP-PTX, respectively. In addition, TEM photograph showed that PC-NP-PTX was spherical particles with regular sizes (Figure 2(A,B)). The EE of NP-PTX, Pep-NP-PTX, CREKA-NP-PTX and PC-NP-PTX was 84.8 ± 2.3%, 83.2 ± 3.2%, 82.4 ± 4.7% and 80.6 ± 3.2%, respectively, with a LC of 4.4 ± 0.1%, 3.6 ± 0.2%, 3.8 ± 0.4% and 3.4 ± 0.5%, respectively.

### *In vitro* PTX release profiles of nanoparticles

PTX release evaluation was performed in PBS at pH 7.4 and 5.0. As shown in Figure 2(C,D), all the nanoparticles released PTX in a biphase manner. After the initial burst release (about 35–40%) for about 12 h, the release rate of PTX slowed down. After 72 h, the cumulative PTX release from NP, Pep-NP, CREKA-NP and PC-NP reached 79.89 ± 1.61%, 73.10 ± 2.88%, 78.44 ± 0.6% and 79.03 ± 1.78%, respectively, in pH 5.0 PBS. The release behavior was similar in pH 7.4 PBS with an accumulative PTX release of 77.50 ± 0.25%, 67.17 ± 0.44%, 77.16 ± 1.67% and 76.43 ± 1.26%, respectively. The burst release of PTX might be caused by the diffusion of PTX adsorbed on the surface of nanoparticles. More importantly, neither Pep-1 nor CREKA modification affected the release pattern of PTX.

### Cellular uptake assay

**Figure 3.**
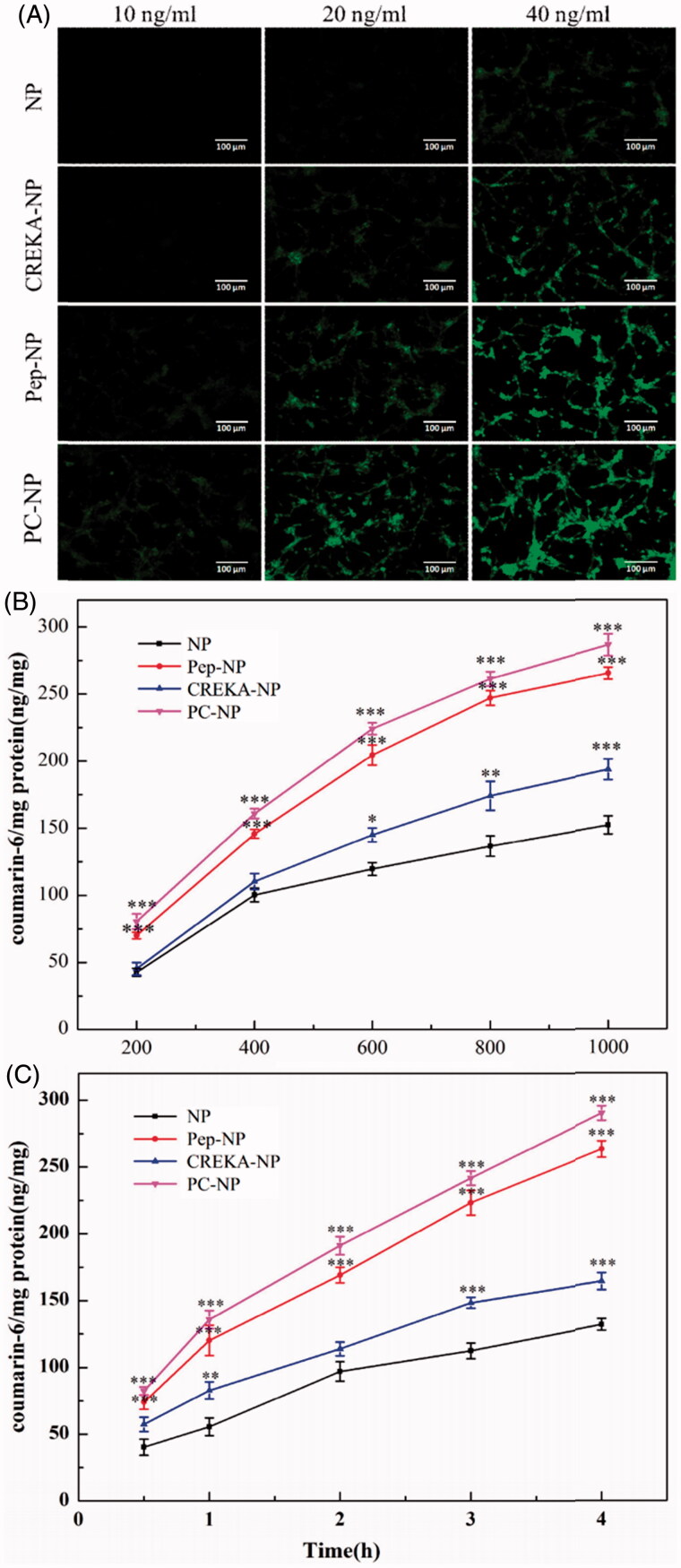
Cellular uptake assay of nanoparticles. (A) Fluorescent microscopy image showing the cellular uptake of coumarin-6-labeled (10, 20 and 40 ng/mL) NP, Pep-NP, CREKA-NP and PC-NP by U87MG cells after incubation at 37 °C for 1 h. Bar: 100 μm. (B) Uptake of various coumarin-6-labeled (200–1000 ng/mL) nanoparticles by U87MG cells at 37 °C for 1 h as determined by HPLC. (C) Uptake of various coumarin-6-labeled (500 ng/mL) nanoparticles by U87MG cells at 37 °C for 0.5–4.0 h as determined by HPLC. Data are presented as mean ± SD (*n* = 3). *p < .05 vs NP, **p < .01 vs NP, ***p < .001 vs NP.

In order to determine whether Pep-1 and CREKA dual modification could increase the glioma targeting effects of PEG-PLGA nanoparticles, the cellular uptake of the coumarin-6-labeled NP, Pep-NP, CREKA-NP and PC-NP by U87MG cells was evaluated using fluorescent microscopy and HPLC. As shown in Figure 3(A), the cellular uptake of all coumarin-6-loaded nanoparticles exhibited a concentration-dependent mode when the concentrations of coumarin-6 ranged from 10 to 40 ng/mL. Furthermore, U87MG cells treated with Pep-1- and/or CREKA-modified nanoparticles emitted stronger fluorescence than the peptide-free nanoparticles. Quantitative analysis results, as shown in Figure 3(B,C), indicated that the cellular uptake of various nanoparticles in U87MG cells displayed a concentration- and time-dependent mode, and was obviously enhanced by Pep-1 conjugation. The fluorescence intensity of Pep-NP and PC-NP was significantly higher than that of NP at all tested concentrations and at each tested time points. Taken together, qualitative and quantitative analysis confirmed that the conjugation of Pep-1 to the surface of PEG-PLGA nanoparticles enhanced the cellular uptake of nanoparticles, highly probably by IL-13Rα2-mediated endocytosis. CREKA modification also led to an increased cellular uptake of the nanoparticles, although to a lesser extent than Pep-1. This could be explained by the alkaline amino acid residues (Arg and Lys) of CREKA, which are positively charged in physiological conditions, thereby adhering to the negatively charged surface of U87MG cells.

### *In vitro* cytotoxicity assay

**Figure 4.**
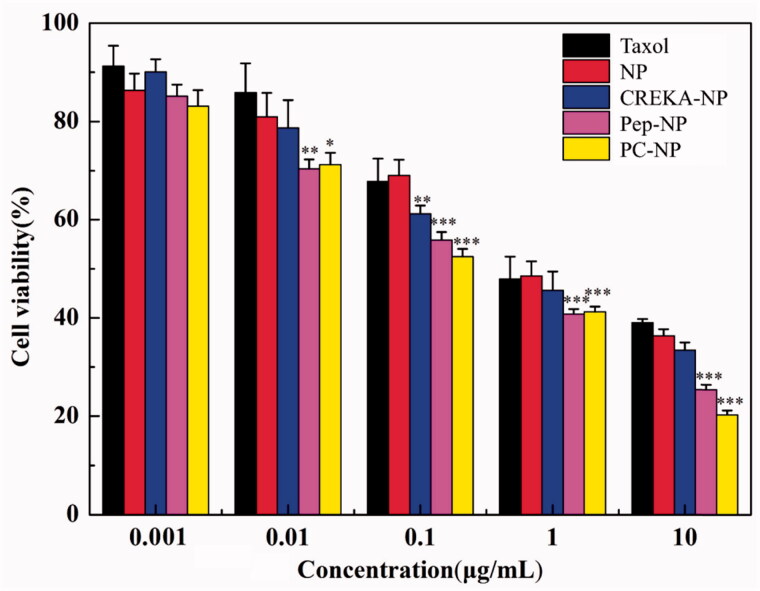
Cytotoxicity studies of Taxol^®^, NP, Pep-NP, CREKA-NP and PC-NP in U87MG cells after incubation for 48 h (*n* = 6). ***p < .001, **p < .01, *p < .05.

To test whether the increased cellular uptake by peptide modification leads to an improved anticancer activity of NP-PTX. The cytotoxicity of different PTX-loaded nanoparticles on U87MG cells was evaluated by MTT assay, using Taxol^®^ as a positive control (Figure 4). The cytotoxicity of NP-PTX is slightly higher than Taxol^®^ as revealed from the IC_50_ values (1.264 and 1.472 μg/mL, respectively). Pep-1 or CREKA modification further increased the cytotoxicity of NP-PTX with an IC_50_ value of 0.255 and 0.714 μg/mL, respectively. Particularly, Pep-1 and CREKA dual-modified PC-NP-PTX exhibited the highest cytotoxicity (IC_50_ = 0.176 μg/mL) among all the formulations. These results were consistent with cellular uptake ability of coumarin-6-labeled nanoparticles (Figure 3), indicating that Pep-1 and CREKA elevate the cytotoxic effect of NP-PTX through receptor- and adsorption-mediated endocytosis by U87MG cells.

### *In vitro* fibrin clot binding assay

**Figure 5.**
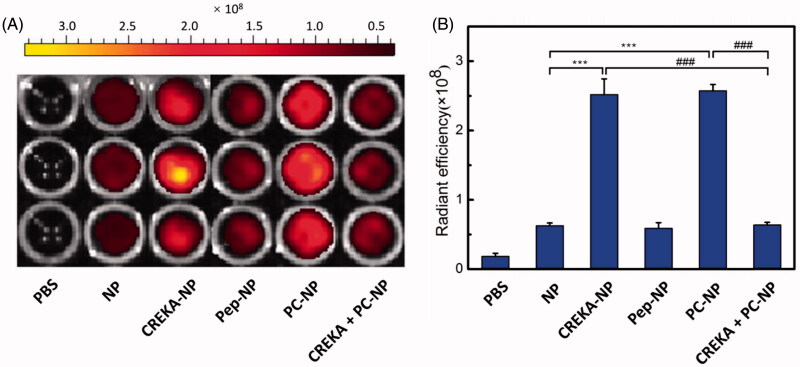
*In vitro* binding of various nanoparticles to FFP clots. (A) IVIS spectrum imaging and (B) corresponding radiant efficacy of FFP clots or CREKA-pretreated FFP clots after incubation with PBS (control) and DiR-labeled nanoparticles (NP, CREKA-NP, Pep-NP and PC-NP). Values were means ± SD, *n* = 3. ***p < .001 compared with NP group, ###*p* < .001 compared with CREKA + PC-NP group.

To evaluate the *in vitro* specific binding potential of various nanoparticles to fibrin clots, FFP clots were incubated with PBS- or DiR-labeled nanoparticles (5 μg/mL DiR) for 30 min and imaged by IVIS spectrum imaging system (PerkinElmer, Maestro, Waltham, MA) (Figure 5). The results showed that the fluorescence intensity of CREKA functionalized nanoparticle (CREKA-NP and PC-NP) was much stronger than that of NP and Pep-NP. Meanwhile, there was no fluorescence in the vehicle control group. Semiquantitative results also showed that the clot fluorescence intensity of NP group was weak, as well as for the Pep-NP group. The clot fluorescence intensities of CREKA-NP and PC-NP were about four-fold higher than that of NP. Furthermore, competition experiments revealed that additional CREKA (500 μg/mL) treatment led to total fluorescence intensity of PC-NP loss due to its competitive binding to fibrin clots. Taken together, our results confirmed that CREKA peptide contributed to the enhanced fibrin clot binding capacity of CREKA-NP and PC-NP, which might facilitate its retention at tumor site *in vivo*.

### *In vivo* organ distribution

**Figure 6.**
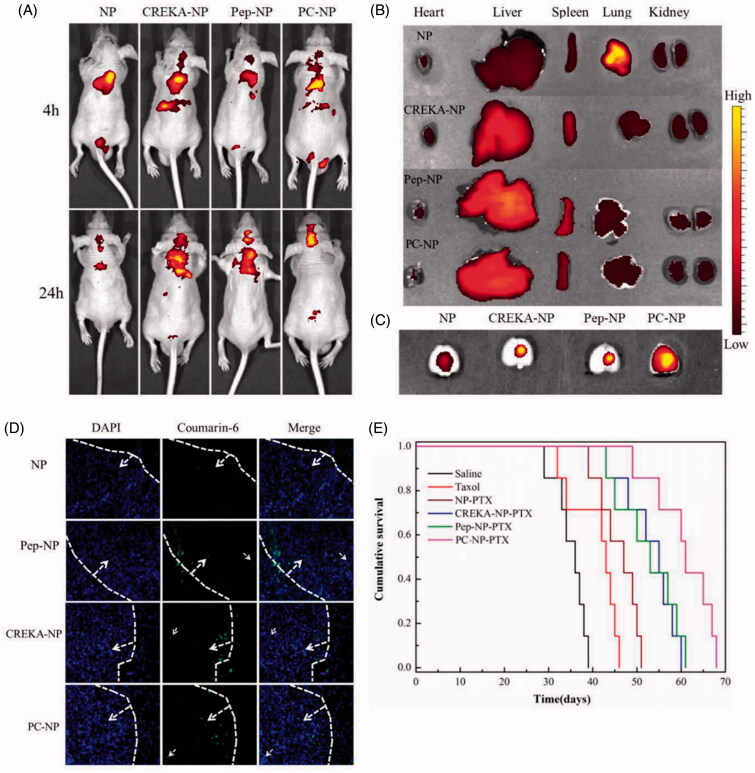
*In vivo* distribution and anti-GBM efficacy of nanoparticles in U87MG glioma-bearing nude mice. (A) *In vivo* real-time fluorescence imaging of U87MG glioma-bearing nude mice administrated with DiR-labeled NP, CREKA-NP, Pep-NP and PC-NP at two different time points (4 h and 24 h). (B) (C) *Ex vivo* fluorescence imaging of organs and brains sacrificed 24 h after treatment. (D) *In vivo* distribution of various coumarin-6-loaded nanoparticles in glioma sections of U87MG glioma-bearing nude mice. Dash lines: border of the glioma. Original magnification: ×20. (E) Kaplan–Meier survival curves for U87MG glioma-bearing mice treated with different PTX formulations at a dose of 10 mg/kg PTX on day 2, 4, 6 and 8 post-implantations.

To evaluate the glioma targeting efficacy of PC-NP *in vivo*, glioma-bearing mice were injected with DiR-labeled NP, Pep-NP, CREKA-NP and PC-NP (0.1 mg/kg) via the tail vein, and real-time *in vivo* imaging was performed. As shown in Figure 6(A), the distribution of DiR-labeled nanoparticles at head–neck domain constantly increased with time and the fluorescence intensity in these regions of Pep-NP, CREKA-NP and PC-NP was clearly higher than that of NP at 4 and 24 h.

To study the distribution of various nanoparticles in brain, heart, liver, spleen, lung and kidney, mice were sacrificed 24 h post-intravenous administration, and the organs were harvested for *ex vivo* imaging. As shown in Figure 6(B), Pep-1 or CREKA modification did not affect the distribution of particles in normal organs, with liver and spleen still be the key eliminating organs (Figure 6(B)). However, the distribution of nanoparticles in brain was significantly increased after CREKA or Pep-1 modification. Only weak fluorescence intensity in brain was observed in NP-treated mice, probably due to the poor EPR effects of NP in glioma brain. Pep-NP, CREKA-NP and PC-NP led to obviously increased fluorescence intensity in brain (Figure 6(C)). Collectively, our results confirmed that the dual peptide modification facilitates the brain accumulation of nanoparticles in GBM-bearing mice.

### *In vivo* glioma distribution

To investigate the distribution of various nanoparticles in glioma, GBM-bearing mice were injected with coumarin-6-labeled nanoparticles (0.2 mg/kg) via the tail vein. One hour later, mice were sacrificed and the brains were harvested for qualitative study by fluorescence microscopy. As shown in Figure 6(D), only a slight distribution of NP in the glioma site was observed. However, clearly higher fluorescence intensities in glioma were found for Pep-1- and/or CREKA-modified nanoparticles. Particularly, PC-NP penetrated deeper into glioma tissue as compared with Pep-NP and CREKA-NP, confirming that the combined effects from Pep-1 and CREKA peptides led to increased penetration and enhanced retention of the nanoparticles in glioma tissue.

### *In vivo* anti-GBM efficacy

To evaluate the *in vivo* antiglioma effects of different formulations, U87MG glioma-bearing mice were divided into six groups and injected with saline, Taxol^®^, NP-PTX, Pep-NP-PTX, CREKA-NP-PTX and PC-NP-PTX via the tail vein, respectively, on days 2, 4, 6 and 8 at a dose of 10 mg/kg PTX. Survival curves for various PTX-loaded formulations were shown in Figure 6(E). Due to the poor glioma targeting capability and low BBTB transporting ability of PTX, only a slightly longer median survival time (MST, Table S2) was observed for PTX-treated group (43 days) compared with the saline group (36 days). NP-PTX prolonged the MST to 47 days due to passive targeting efficiency. However, the rather small microvascular pore size along with the sustained high interstitial fluid pressure in glioma resulted in poor EPR effect, which limited the antiglioma activity of NP-PTX. Covalent binding of Pep-1 or CREKA to the surface of PEG-PLGA nanoparticles further increased the antiglioma activity with a MST value of 53 days and 55 days for Pep-NP-PTX and CREKA-NP-PTX, respectively. Dual modification with Pep-1 and CREKA resulted in the best antiglioma activity as indicated by the longest MST (61 days) of PC-NP-PTX-treated group.

## Conclusions

In summary, we designed and prepared a peptide-mediated nano-DDS by conjugating peptides Pep-1 and CREKA to the surface of PEG-PLGA nanoparticle via a maleimide–thiol reaction, affording PC-NP. Pep-1, a cell-penetrating peptide, could cross the BBTB and target the brain glioma via IL-13Rα2-mediated endocytosis. CREKA, a clot-binding peptide, could target tumor extracellular matrix by binding to fibrin–fibronectin complexes. Therefore, the PC-NP is expected to have improved glioma targeting ability and enhanced retention in tumor. PTX-loaded PC-NP was spherical with a mean particle size of 101.1 ± 2.8 nm and a zeta potential of −25.6 ± 2.5 mV. The release of PTX from PC-NP-PTX was confirmed *in vitro*. The dual modification of PEG-PLGA nanoparticles by Pep-1 and CREKA significantly increased the cytotoxicity of its payload PTX against U87MG cells with an IC_50_ of 0.176 μg/mL. *In vivo* fluorescence imaging confirmed that PC-NP could accumulate effectively and penetrate deeply in GBM tissue. *In vivo* anti-GBM evaluation revealed that PC-NP-PTX exhibited powerful anti-GBM efficacy with the longest MST (61 days) among all the treatments. Taken together, our findings indicated PC-NP as a promising nano-DDS for glioma targeting delivery of anticancer drugs.

## Supplementary Material

IDRD_Xin_et_al-Supplemental_Content.docx
