# Supplementary material for: Glioma and microenvironment dual targeted nanocarrier for improved antiglioblastoma efficacy
Source: Drug Deliv. 2017 Sep 21;24(1):1401–9. doi: 10.1080/10717544.2017.1378940 (PMC8241031; doi:10.1080/10717544.2017.1378940)
Supplement: IDRD_Xin_et_al-Supplemental_Content.docx [file IDRD_A_1378940_SM3378.docx]

**Glioma and microenvironment Dual Targeted Nanocarrier for Improved Antiglioblastoma Efficacy**

Xiuzhen Wang^a,b#^, Qing Zhang^b#^, Lingyan Lv^b^, Junjie Fu^b^, Yan Jiang^b^, Hongliang Xin^b^*, Qizheng Yao^a^*

a Department of Medicinal Chemistry, School of Pharmacy, China Pharmaceutical University, Nanjing 210009, China

b School of Pharmacy, Nanjing Medical University, Nanjing 211166, China

^#^These authors contributed equally to this manuscript.

Corresponding author:

Hongliang Xin (Tel.: +86-25-86868467, Fax: +86-25-86868467, E-mail addresses: xhl@njmu.edu.cn).

Qizheng Yao (Tel.: +86-25-86634730, Fax: +86-25-86634730, E-mail addresses: [qz_yao@163.com](mailto:qz_yao@163.com)).

**Table of contents**

Synthesis and characterization of Pep-PEG-PLGA and CREKA-PEG-PLGA S2

Characterizations of NP-PTX, Pep-NP-PTX, CREKA-NP-PTX and PC-NP-PTX S5

*In vivo* effects of PTX formulations on intracranial U87MG glioma mice model S6

# Synthesis and characterization of Pep-PEG-PLGA and CREKA-PEG-PLGA

Pep-1(Cys-Gly-Glu-Met-Gly-Trp-Val-Arg-Cys) and CREKA (Cys-Arg-Glu-Lys-Ala) were synthesized by the GL Biochem Co., Ltd. (Shanghai, China). MePEG-PLGA and Maleimide-PEG-PLGA were obtained from Daigang Biomaterial Co., Ltd. (Jinan, China). PTX was purchased from Zelang Medical Technology Co., Ltd. (Nanjing, China). All other chemical reagents were analytical grade.

Pep-PEG-PLGA was synthetized and characterized as described previously (*Biomaterials* **2014**, *35*, 5897–5907). CREKA-PEG-PLGA was synthesized through Michael addition between the cysteine residue of CREKA peptide and the maleimide moiety in Maleimide-PEG-PLGA. In brief, a solution of Maleimide-PEG-PLGA (20 mg) in DMF (2 mL) was added slowly into distilled water (9 mL) under magnetic stirring (650 rpm) during 5 min at room temperature, forming the suspension of Maleimide-PEG-PLGA nanoparticles. CREKA (1 mg) in PBS (pH 7.4, 1 mL) was then added into the above suspension at room temperature. The mixture was stirred for a further 6 h under nitrogen atmosphere. Excess CREKA and DMF were removed by dialysis against distilled water, and CREKA-PEG-PLGA was obtained by freeze drying.

The formation of CREKA-PEG-PLGA was confirmed by ^1^H NMR (Figure S1). As shown in Figure S1A, signals located at 1.54–1.59, 4.67–4.91 and 5.17–5.22 ppm represent PLAG. The peak at 3.64 ppm belongs to methylene protons in PEG. The peak at 6.7 ppm, a characteristic signal of the maleimide group in Mal-PEG-PLGA, disappears in the spectrum of CREKA-PEG-PLGA (Figure S1B). At the same time, new signals at 0.85,1.25 and 2.17 ppm corresponding to CREKA are detected in Figure S1B. The above results confirmed that the CRKEA peptide was successfully conjugated to Maleimide-PEG-PLGA.

The FTIR spectra of CREKA-PEG-PLGA was shown in Figure S2. A C=O stretching vibrational absorption at 1759 cm^−1^ is attributable to the maleimide moiety. There are two characteristic absorptions of peptide fragments located around 3414 cm^−1^ and at 1647 cm^−1^, which are attributed to NH or OH stretching vibration and C=O stretching absorption, respectively.


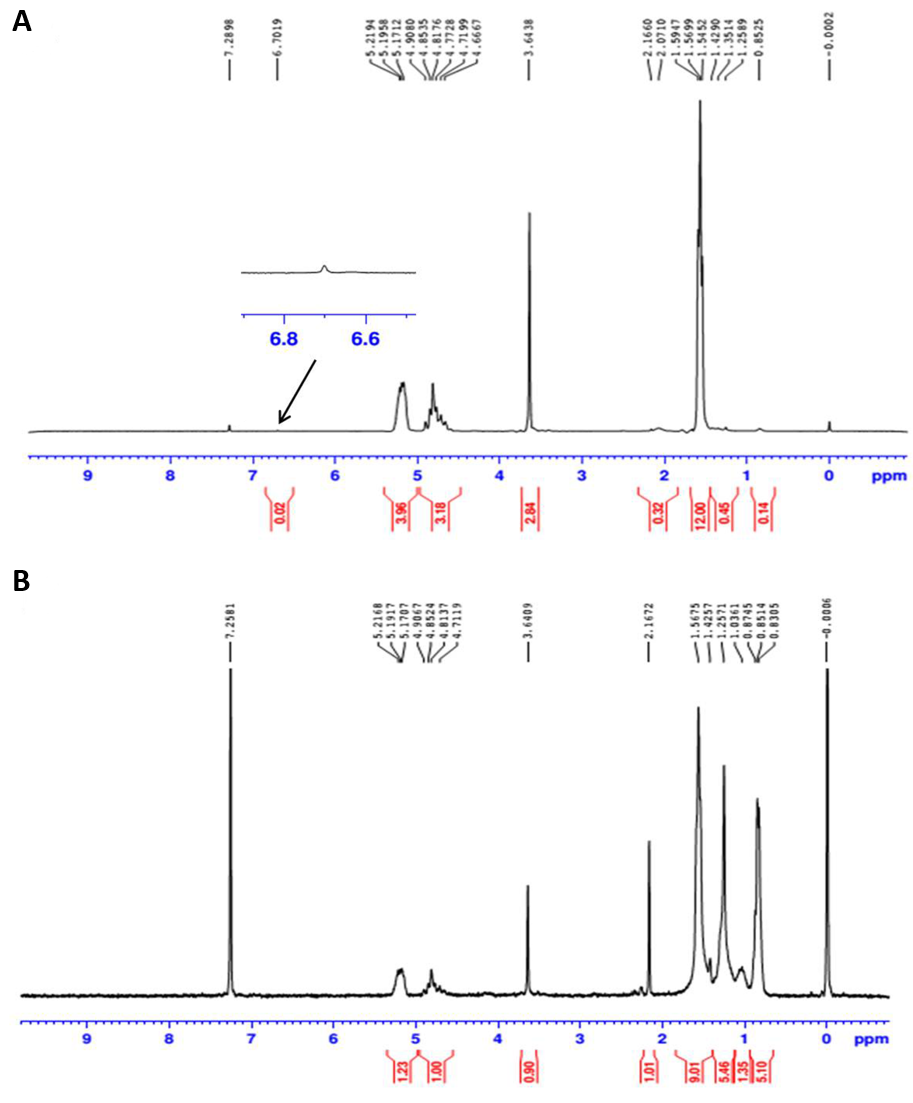


**Figure S1.** ^1^H NMR spectra of Mal-PEG-PLGA (A) and CREKA-PEG-PLGA (B) in CDCl_3_.


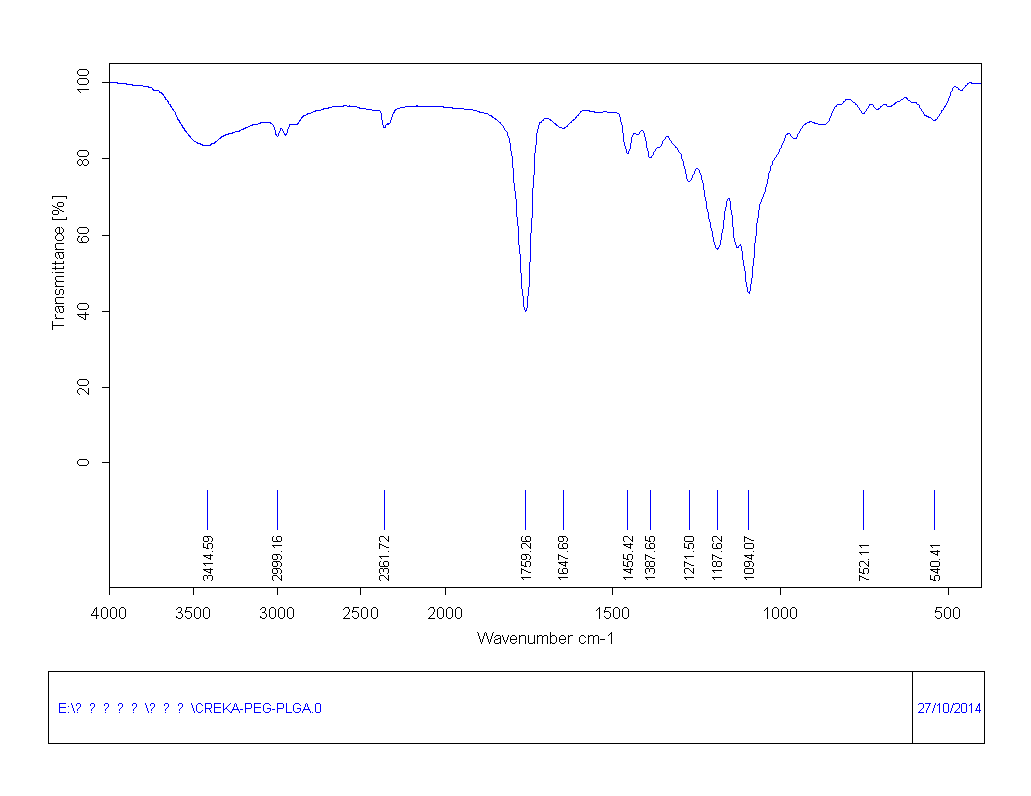


**Figure S2.** FTIR spectra of CREKA-PEG-PLGA.

# Characterizations of NP-PTX, Pep-NP-PTX, CREKA-NP-PTX and PC-NP-PTX

**Table S1.** Characterizations of various PTX-loaded nanoparticles.

| **Formulation** | **Particle size**  **(nm)**^a^ | **Zeta Potential**  **(mV)**^a^ | **EE%**^a^ | **LC%**^a^ |
| --- | --- | --- | --- | --- |
| NP-PTX | 89.3 ± 1.2 | -32.5 ± 1.2 | 84.8 ± 2.3 | 4.4 ± 0.1 |
| Pep-NP-PTX | 93.2 ± 2.4 | -33.6 ± 1.3 | 83.2 ± 3.2 | 3.6 ± 0.2 |
| CREKA-NP-PTX | 95.8 ± 4.2 | -20.3 ± 3.1 | 82.4 ± 4.7 | 3.8 ± 0.4 |
| PC-NP-PTX | 101.1 ± 2.8 | -25.6 ± 2.5 | 80.6 ± 3.2 | 3.4 ± 0.5 |
| ^a^Data are represented as mean ±SD (n = 3). | | | | |

# *In vivo* effects of PTX formulations on intracranial U87MG glioma mice model

**Table S2.** *In vivo* effects of PTX formulations on intracranial U87MG glioma mice model (n = 8)

| **Groups** | **Dose**  **(mg/kg)** | **MST^a^**  **(days)** | **Median**  **(days)** | ***vs***  **Saline^b^** | ***vs***  **Taxol^®b^** | ***vs***  **NP^b^** | ***vs***  **CREKA-NP^b^** | ***vs***  **PEP-NP^b^** |
| --- | --- | --- | --- | --- | --- | --- | --- | --- |
| Saline | — | 35.1 ± 1.3 | 36 | — | — | — | — | — |
| Taxol^®^ | 10 | 40.9 ± 2.1 | 43 | * | — | — | — | — |
| NP-PTX | 10 | 46.0 ± 1.7 | 47 | *** | * | — | — | — |
| CREKA-NP-PTX | 10 | 53.1 ± 2.3 | 55 | *** | ** | * | — | — |
| PEP-NP-PTX | 10 | 52.6 ± 2.6 | 53 | *** | ** | * | > 0.05 | — |
| PC-NP-PTX | 10 | 60.7 ± 2.6 | 61 | *** | *** | ** | * | ** |
| ^a^MST: mean survival time  ^b*^P < 0.05, ^**^P < 0.01, ^***^P < 0.001 of log-rank analysis | | | | | | | | |
